# Effects of SiO_2_ content on the nanomechanical properties of CoCrPt-SiO_2_ granular films

**DOI:** 10.1038/s41598-019-54093-2

**Published:** 2019-11-26

**Authors:** Youfeng Zhang, Ahmad Shakil, Hongbo Wang, Xinwei Li, Huan Tang, Andreas A. Polycarpou

**Affiliations:** 1 Department of Mechanical Engineering, Texas A&M University, College Station, TX 77843 USA; 2 Recording Media Operation, Seagate Technology LLC, 47488 Kato Road, Fremont, CA 94538 USA

**Keywords:** Structural properties, Mechanical engineering

## Abstract

CoCrPt material is used for perpendicular magnetic recording media due to its high magneto-crystalline anisotropy that brings good thermal stability on the media. The addition of SiO_2_ between the CoCrPt grains offers benefits including lower noise and better thermal stability. It has been reported that the SiO_2_ content has strong effects on the media’s recording performance such as coercivity, anisotropy and noise. In this work, we focus on studying the effects of the SiO_2_ content on the nanomechanical properties of the media which are critical for the head-disk interface reliability. Variations of these properties with SiO_2_ content provide guidelines for optimum designs considering both recording and mechanical interface performance.

## Introduction

Perpendicular magnetic recording (PMR) technology was a breakthrough for achieving area densities of data storage higher than 100 Gb/in^2^ in the hard disk industry drive (HDD) industry. Compared to the previous technology of longitudinal magnetic recording (LMR), the key difference of PMR is that the grain structure and the magnetic orientation of the stored data of PMR media is granular/perpendicular instead of longitudinal. This new media structure feature brings advantages including improved thermal stability, improved signal-to-noise ratio (SNR) due to better grain separation and uniformity, and better writability due to stronger head fields and better magnetic alignment of the media^1–3^. The most common material used for the PMR magnetic layer is CoCrPt-SiO_2_ composite material that has CoCrPt granular grains/islands as data storage media and SiO_2_ between grains as segregation material. The addition of SiO_2_ is important and its content has significant effects on media recording performance. An increase of SiO_2_ decreases the magnetic grain size, causing very high magnetic anisotropy. Increasing SiO_2_ content also increases magnetic coercivity due to grain isolation, therefore increases data storage capacity and reduces noise^4,5^. A SiO_2_ content of 11–14% reportedly gives optimum recording performance^4^. Even for the promising next-generation PMR technologies, the so-called heat assisted magnetic recording (HAMR)^6^, and bit-patterned media recording (BPMR)^7^, SiO_2_ is still the primary choice as the segregation material between media grains^7,8^. L10-FePt is also proposed as a promising magnetic media due to very high magnetic anisotropy but the switching magnetic field needs to be very high, which cannot be afforded by the available write recording heads, and further improvements are needed before implementation^9^.

Besides recording properties, nanomechanical and nanotribological performances of HDDs are also very important for reliability. Studies show that head-media spacing of less than 6.5 nm is required to achieve 1 Tb/in^2^ magnetic areal density in HDDs^10^. As the media rotates at a very high speed, the head can accidently come into contact with the media and may scratch or wear out the surface. Although diamond like carbon (DLC), also known as carbon overcoat (COC), is commonly used to protect the media, in the worst case scenario, scratch and wear may lead to contact induced damage of the media, causing demagnetization and loss of data from the media^11–13^. Therefore, the investigation of the effects of SiO_2_ content on the nanomechanical and nanotribological properties of the media is critical, but has not been conducted systematically. In this work, we measure the nanomechanical and nanotribological properties of three media samples with different SiO_2_ contents. The dependence of the properties on the SiO_2_ content are quantified by growth constants obtained from curve-fitting of the experimental results. These relationships can be used for design of the media composite thin films, with concerns on HDI nanomechanics, nanotribology, and reliability, regardless of the material type for the magnetic grains. The ultimate goal is to provide guidelines to optimize the material design in terms of both recording and mechanical performance.

## Samples and Instrumentation

### Sample description

**Figure 1:**
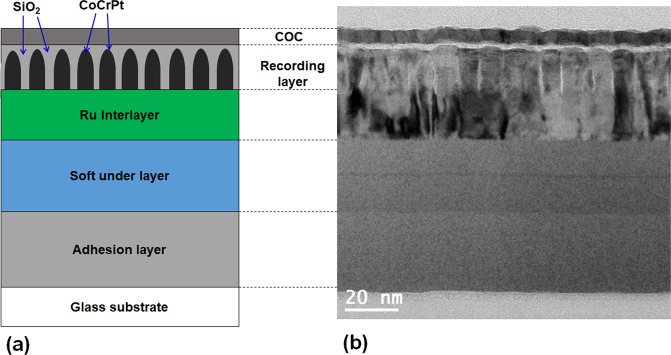
(**a**) Schematic of a full-stack perpendicular recording hard disk (cross-section view), (**b**) Cross section TEM image of a perpendicular recording hard disk.

Figure 1 shows a Transmission Electron Microscopy (TEM) cross-sectional view of a typical PMR magnetic disk with multiple layers of solid films. On the very top, there is a carbon overcoat (COC) with a thickness of ~2 nm to protect the recording media layer from mechanical wear and chemical corrosion. The media layer is a 14-nm thick CoCrPt-SiO_2_ composite. Below the media layer, there is a Ru-based interlayer for exchange breaking and inducing texture growth on the media layer. Below the interlayer, there is a magnetic soft under layer (SUL) to help writing the magnetic data. Except for the COC layer that is normally fabricated using plasma enhanced chemical vapor deposition (PECVD), the other films are deposited by magnetron sputtering. The CoCrPt-SiO_2_ film media were deposited on 2.5-in glass-substrate disks by a co-sputtering method with Co, Pt, Cr targets using an UHV-magnetron sputtering system. A Ru film was used as the seed layer. The composition of SiO_2_ can be varied by controlling the deposition rate. The standard film thickness of the CoPtCr layer was 20 nm.

**Figure 2:**
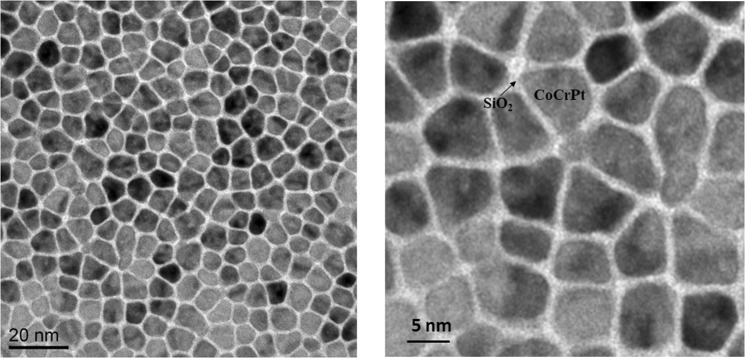
TEM planar views of the magnetic medium layer.

Figure 2 depicts planar views of the CoCrPt-SiO_2_ media layer under TEM. The dark grains are CoCrPt with size of about 10 nm and the white segregation material between the grain boundaries is SiO_2_. Samples with three different SiO_2_ contents, 0%, 10.75% and 21.5%, were collected directly from the vendor. All other solid films remain same in all samples for the study.

### Instrumentation and experimental setup

Shallow nanoindentation experiments utilized a 1-axis microelectromechanical systems (MEMS) transducer (Hysitron xProbe^®^) installed with a cube corner probe with a tip radius of ~50 nm^14^. The root-mean-square (RMS) force resolution is ~5 nN and the RMS displacement resolution is 0.05 nm. Utilization of such a sensitive transducer and a sharp probe is to ensure that the transducer is capable of capturing the elastic and plastic behaviors of the composite thin films without significant substrate effects. Calibration experiments are performed on a standard fused quartz sample with known mechanical properties (elastic modulus = 69.6 ± 5% GPa, hardness = 9.25 ± 10% GPa). It is shown that the transducer is capable of obtaining accurate nanomechanical properties for contact depths down to ~2 nm.

The present nanoscratch experiments employ a 2-axis standard transducer using a TI Premier (Hysitron^®^) with an RMS force resolution of 0.045 µN and an RMS displacement resolution of 0.22 nm. The probe for the scratch experiments is conospherical with a tip radius of 350 nm.

## Results and Discussion

### Nanoindentation

**Figure 3:**
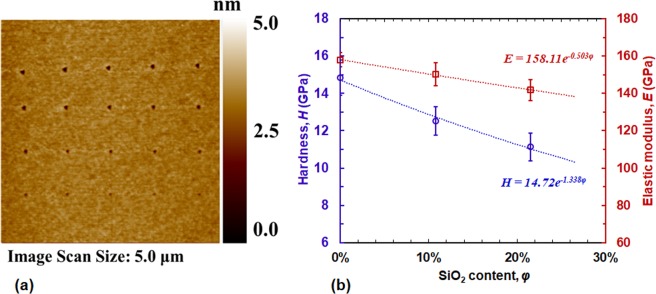
(**a**) SPM residual image using indentation loads varying from 30 μN to 11 μN; (**b**) Measured mechanical properties varying with SiO_2_ content and their curve-fitted relations: *E* = *158.11e*^*−0.503φ*^ and *H* = *14.72e*^*−1.338φ*^.

For each sample, we performed 20 nanoindentation experiments with peak load varying from 30 μN down to 11 μN. The load function is of trapezoidal shape: 5 seconds loading, 2 seconds holding and 5 seconds unloading. The corresponding contact depths were limited to less than 8 nm to avoid substrate effects from the intermediate layer. Figure 3(a) shows residual scanning probe microscopy (SPM) images on the sample with 10.75% SiO_2_. The indentation experiments were set up as a 4 × 5 matrix with a spacing of 1 μm between adjacent indentations.

The nanoindentation technique measures the reduced elastic modulus that is calculated from the unloading stiffness and the contact area: ${E}_{r}=\frac{S}{2\beta }\sqrt{\frac{\pi }{A}}$^15^, where *S* is the slope of the unloading curve or called unloading stiffness, *β* is a correction factor and equal to 1 for the current tip. In addition, the hardness *H* can be determined by the mean contact pressure under the indenter, i.e., $H=\frac{{P}_{max}}{A}$, where *P*_*max*_ is the peak indentation load^15^. Figure 3(b) plots the extracted average elastic modulus and hardness values for the three samples, measured from nanoindentation. It can be seen that the addition of SiO_2_ reduces both elastic modulus and hardness of the composite film. The decreasing trend may be attributed to reduction in grain size with increase in SiO_2_ content. Reduction in grain size indicates increase in grain boundary size filled with SiO_2_. As SiO_2_ is softer than CoFePt, the overall composite film may become softer with increase in SiO_2_ content, causing reduction in both elastic modulus and hardness. Curve-fitting using exponential functions shows that the growth constant of the elastic modulus is −0.503 while that of the hardness is −1.338: The hardness decreases more rapidly than the elastic modulus as the SiO_2_ content increases.

The elastic modulus and hardness values originate from elastic and plastic behaviors of the material being pressed by the indenter. It should be noted that hardness is defined as the mean contact pressure when a rigid indenter is pressed on a deformable body. Hardness is an indicator of the material’s yield strength, however, not a direct fundamental material property that can be used in constitutive relations. The relationship proposed by Tabor relates indentation hardness *H* with the yield strength *σ*_y_ through a coefficient of 3, i.e., *H* = 3*σ*_*y*_^16^. This relation makes good predictions for plastic metal materials (*E/σ*_*y*_>>1) such as bulk copper and steel. However, it is found that for materials with small *E/σ*_*y*_ ratios, the coefficient for *H/σ*_*y*_ is only about 1.7^17^. In addition, regression formulas between indentation/scratch hardness and the yield strength were obtained from finite element simulations^18,19^. Johnson proposed a more generic relationship considering indenter geometry and material properties^20^:

1 $$\frac{H}{{\sigma }_{y}}=\frac{2}{3}[1+ln(\frac{1}{3}\frac{E}{{\sigma }_{y}}\,\tan \,\beta )]$$

where *β* is the equivalent angle between the indenter and the sample surface plane, and is equal to 47.7° for the cube corner tip used in the present study. This relationship has been validated by correlating finite element simulations with nanoindentation testing data on sub-20 nm thin films^21^.

**Figure 4:**
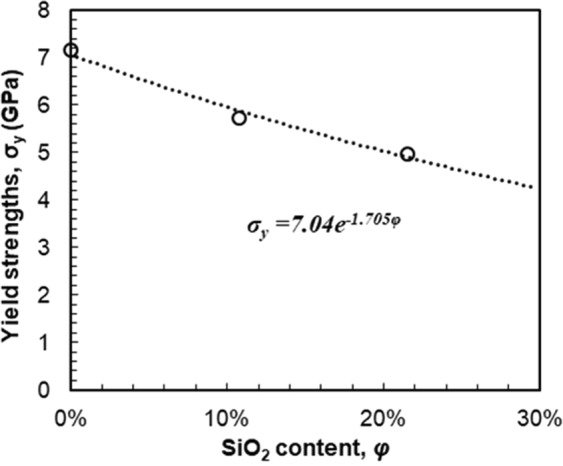
Variation of the yield strength with SiO_2_ content and curve-fitted relationship, *σ*_*y*_ = *7.04e*^*−1.705φ*^.

Figure 4 plots the yield strength values of the three samples calculated by Eq. (1) and curve-fits the variation with respect to the SiO_2_ content. The yield strength decreases with increasing SiO_2_ content with a relationship fitted by *σ*_*y*_ = *7.04e*^*−1.705φ*^. The growth constant is −1.705 for the yield strength, which is larger than that for hardness. With the addition of SiO_2_, the composite film is more readily subject to plastic yielding.

### Nanoscratch

Nanoscratch has been widely applied to characterize contact and tribological behavior of thin films and coatings under sliding contact, including friction and scratch/wear resistance^22,23^. Unlike nanoindentation that measures mechanical properties, nanoscratch is capable to simulate working (sliding) conditions and measure the tribological behavior of the sample surface under sliding contact with the probe. This is especially so in the HDD industry, where nanoscratch is frequently employed to evaluate wear resistance and durability of the disk under impact from particles found in the HDD or impact with the recording head.

#### Experimental setup

**Figure 5:**
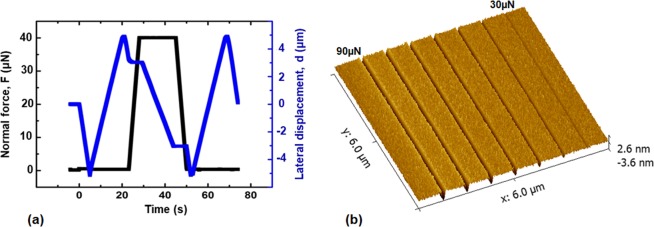
(**a**) Load function for nanoscratch experiments, (**b**) AFM image of the residual grooves after scratch experiments using different normal forces.

The load function used in the present nanoscratch measurements is shown as in Fig. 5(a). The scratch test includes three steps: (1) pre-scan step from 0 to 20 s for data correction with a light contact force of 2 µN to remove misalignment of the normal displacement due to tilt or gradient of the sample surface; (2) loading step from 20 s to 50 s for the tip to scratch laterally the sample surface with a constant normal load; and (3) retrace step for the tip to scratch backwards with a light contact force of 2 µN to measure the residual depth of the groove. For each sample, we performed seven experiments with the scratch load varying from 30 µN to 90 µN with an increment of 10 µN. There is a spacing of 0.75 µm between two adjacent nanoscratch tracks. Figure 5(b) displays the atomic force microscopy (AFM) image of a media sample after a set of scratch experiments that was scanned using a sharp AFM tip with a tip radius of 10 nm. The groves from left to the right correspond to residual wear tracks after completion of the nanoscratch experiments with a normal load varying from 90 µN to 30 µN. The depths of the wear tracks will be compared and discussed in a later section.

#### Coefficient of friction (COF)

**Figure 6:**
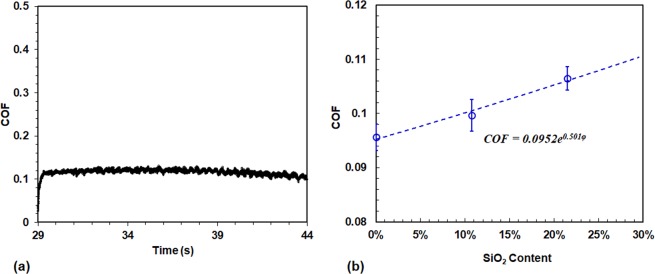
COF from nanoscratch experiments (**a**) typical *in-situ* COF at a load of 30 µN; (**b**)Summary of COF data for the three samples and curve-fitted relation with SiO_2_, COF = 0.0952*e*^*0.501φ*^.

The COF is an important parameter to quantify levels of frictional resistance at contacting/ sliding interfaces. Figure 6(a) shows the COF data at the scratch load of 30 µN. With a constant scratch load, the friction resistance from the sample surface is also nearly constant. The average COFs for the three samples are plotted in Fig. 6(b). The COF values for scratch load from 30 μN to 90 μN are similar, which is also indicated by small data deviation. The average COF for the three samples increases slightly with increasing SiO_2_ content, with a growth constant of 0.501.

The simple theory proposed by Bowden and Tabor can be used for interpretation of the COF data^24^. The theory attributes the COF to two basic components that are additive: deformation friction ${\mu }_{def}$ and adhesive friction $\,{\mu }_{adh}$. At the initial stage of contact, the major contributor to the total friction is adhesion between the two surfaces arisen from Van der Waals forces. As the contact force increases, the major source of friction is resistance of the softer material to ploughing of a hard asperity (the diamond probe in this case). The deformation friction coefficient for a rigid sphere sliding on a soft surface is linear with the square root of the ratio of the *in-situ* depth to the spherical tip radius, $0.6\sqrt{{h}_{i}/R}$^25^. The *in-situ* depth is the maximum scratch depth measured in the loading step and includes both elastic and plastic deformation.

The three samples share the same COC coating and thus their adhesive friction components should be the same. Therefore, the difference on their COFs is due to the deformation friction that is determined by the *in-situ* scratch depth *h*_*i*_. When the sample is under scratching by the probe, the elastic deformation is the major contributor of the total deformation. For example, even for the sample with 21.5% SiO_2_ that shows the highest residual depth (2.75 nm) at a load of 90 μN, its corresponding *in-situ* depth is about 11 nm. For the other two samples, the contribution from the plastic deformation (residual depth) to the total *in-situ* depth is much smaller as their yield strength is higher. Thus, the contribution from elastic deformation is dominant to the *in-situ* depth. That is, the COF values of the three samples are determined by their elastic moduli shown in Fig. 3. As the SiO_2_ content increases, the elastic modulus gets smaller and the elastic deformation under the same load larger. This correlation is verified by curve-fitting, based on the exponential function in Fig. 6(b). The COF reports a growth constant of 0.501 with SiO_2_ content, which is very close to that of elastic modulus (0.503), but lower than for hardness (1.338), and yield strength (1.705).

#### Residual scratch depths and wear rates

**Figure 7:**
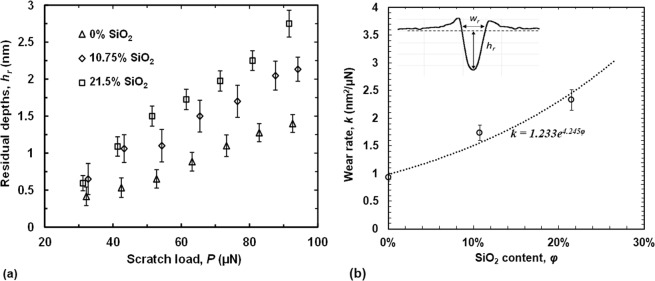
(**a**) *In-situ* residual scratch depths versus scratch load for the three samples with different SiO_2_ contents (**b**) Calculated wear rates and its relationship with SiO_2_ content, *k* = *1.233e*^*4.245φ*^.

The residual depths are the maximum depth values of the plastically deformed grooves after scratching by the diamond probe. Figure 7(a) summarizes the residual depths from the nanoscratch experiments using different normal loads. Each data point reports the average and the standard deviation from three separate experiments. At the minimum load of 30 µN, plastic deformation of the COC coating is more dominant than that in the media layer, so the residual depths are similar. With increase of the scratch load, the scratch depth increases and the probe begins to detect properties of the media layer that is relatively softer and less wear resistant. The difference between the samples with 10.75% SiO_2_ and 21.5% SiO_2_ does not become significant until the scratch load increases to about 50 µN. After that, under the same load, the sample with 21.5% SiO_2_ reports the largest residual wear depths and the one with no SiO_2_ reports the lowest. With an increase in SiO_2_ content, hardness and yield strength decrease, as shown in Figs. (3) and (4), causing an increase of the residual depth. At the load of 90 μN, the residual depth of the 21.5% SiO_2_ sample is almost two times that for the 0% SiO_2_ sample.

To compare the wear resistance of the three samples, in the present work we calculate the specific wear rate, or wear coefficient defined by Archard^26^:

2 $$k=\frac{{V}_{w}}{Fs}=\frac{{h}_{r}{w}_{r}}{2F}$$

where *V*_*w*_ is the wear volume, *F* is the normal load and *s* is the sliding distance. For the constant load scratch experiments in the present work, the wear track can be approximated as a triangular prism and its volume is calculated by ${V}_{w}={h}_{r}{w}_{r}s/2$. Only data points for load values from 50 μN to 90 μN are used in the calculation to avoid effects from the COC. Wear rates of the three samples are plotted with versus SiO_2_ content, as shown in Fig. 7(b). Similar to the nanomechanical properties, the wear rate can be curve-fitted using an exponential function: $k=1.233{e}^{4.245\varphi }$. This growth constant is more than two times the growth constant for the yield strength, indicating a much greater effect of the SiO_2_ content on the tribological properties under scratch.

### Nanoscratch under TEM

**Figure 8:**
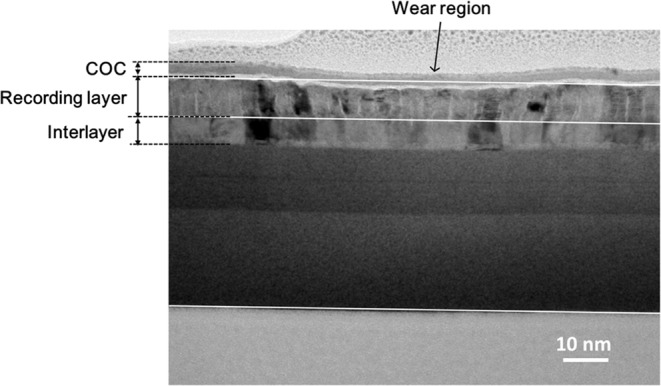
TEM cross sectional view of the wear track after a scratch using 100 µN force.

For nanoscratch experiments, it is important to minimize the substrate effect to ensure that the probe detects the tribological behavior of the layer of interest. As we are dealing with a multilayered system and the media layer is an anisotropic composite, it is necessary to capture material deformation and failure of different layers. We performed a scratch experiment in a Transmission Electron Microscope (TEM) to observe the cross-sectional view of the multilayered system subjected to the nanoscratch. Figure 8 shows a cross sectional view of the wear track after a nanoscratch experiment at a normal load of 100 µN using a probe with a tip radius of 250 nm (10.75% SiO_2_ sample). The scratch direction is perpendicular (out of plane) to the image. The wear track or the groove is formed due to the plastic deformation in comparison with the straight lines representing boundaries before being scratched. The maximum wear depth caused by such a high-pressure scratch is measured to be 2.54 nm. As shown in the TEM image, almost all the plastic deformation is confined within the CoCrPt-SiO_2_ media layer. When under a lateral scratch, the anisotropic granular structure makes the thin film more readily plastically deformed. The grain tilting in the damaged region reportedly results in demagnetization of the media^11^.

## Conclusions

The present study investigates the effects of the SiO_2_ content on the nanomechanical and nanotribological properties of a 14-nm nanocomposite thin films in magnetic storage media. Through nanoindentation and nanoscratch experiments using state-of-the-art nanomechanical instrumentation, it is found that the SiO_2_ content affects the nanomechanical and nanotribological properties of the thin film and the following conclusions could be drawn.

(1) The measurement of the nanomechanical properties shows that the composite thin film becomes more compliant and softer with the addition of SiO_2_, segregated between media grains. The elastic modulus decreases slightly with SiO_2_ content, with a decay constant of 0.503. In contrast, plastic behavior including hardness and yield strength reduces more rapidly with SiO_2_ content, with decay constants of 1.338 and 1.705, respectively.

(2) From nanoscratch experiments, it is observed that the COF increases slightly with SiO_2_ content. The growth constant is similar to the decay constant of the elastic modulus, due to the dominant fraction of the elastic deformation in the total *in-situ* scratch depth. The wear rate is the most direct measure for wear resistance of the sample to external contact/scratch. It increases rapidly with SiO_2_ content, with a growth constant of 4.245. The addition of SiO_2_ into the composite makes it more readily to be worn by scratching. Attention should be paid to the wear rate when the SiO_2_ content is adjusted for optimized HDD media designs. An 11% of SiO_2_ content, it is suggested to achieve both wear resistance and magnetic performance that is optimum in the range of 11%~14%^4^.

## References

[1] Oikawa T (2002). Microstructure and magnetic properties of CoPtCr-SiO_2_ perpendicular recording media. IEEE Trans. Magn..

[2] Piramanayagam SN (2007). Perpendicular recording media for hard disk drives. J. Appl. Phys..

[3] Kaitsu I, Inamura R, Toda J, Morita T (2006). Ultra high density perpendicular magnetic recording technologies. Fujitsu Sci. Tech. J..

[4] Inaba Y (2004). Optimization of the SiO_2_ content in CoPtCr-SiO_2_ perpendicular recording media for high-density recording. IEEE Trans. Magn..

[5] Xu Y (2001). Structural and magnetic properties of HCP-CoCrPt-SiO_2_ granular media. J. Magn. Magn. Mater..

[6] Weller D (2014). A HAMR Media Technology Roadmap to an Areal Density of 4 Tb/in^2^. IEEE Trans. Magn..

[7] Albrecht TR (2015). Bit-Patterned Magnetic Recording: Theory, Media Fabrication, and Recording Performance. IEEE Trans. Magn..

[8] Ju G (2015). High Density Heat-Assisted Magnetic Recording Media and Advanced Characterization—Progress and Challenges. IEEE Trans. Magn..

[9] Varvaro G, Laureti S, Fiorani D (2014). L10 FePt-based thin films for future perpendicular magnetic recording media. J. Magn. Magn. Mater..

[10] Gui J (2003). Tribology challenges for head-disk interface toward 1 Tb/in^2^. IEEE Trans. Magn..

[11] Xu J, Furukawa M, Nakamura A, Honda M (2009). Mechanical Demagnetization at Head Disk Interface of Perpendicular Recording. IEEE Trans. Magn..

[12] Ovcharenko A, Yang M, Chun K, Talke FE (2010). Simulation of Magnetic Erasure Due to Transient Slider-Disk Contacts. IEEE Trans. Magn..

[13] Liu Y, Xiong S, Lou J, Bogy DB, Zhang G (2014). Quantitative relationship between contact stress and magnetic signal strength in perpendicular recording media. J. Appl. Phys..

[14] Zhang Y, Oh Y, Stauffer D, Polycarpou AA (2018). A microelectromechanical systems (MEMS) force-displacement transducer for sub-5 nm nanoindentation and adhesion measurements. Rev. Sci. Instrum..

[15] Oliver WC, Pharr GM (1992). An improved technique for determining hardness and elastic modulus using load and displacement sensing indentation experiments. J. Mater. Res..

[16] Tabor, D. *The Hardness of Metals* (Oxford University Press, 2000).

[17] Cheng YT, Cheng CM (1998). Scaling approach to conical indentation in elastic-plastic solids with work hardening. J. Appl. Phys..

[18] Lee KM, Yeo CD, Polycarpou AA (2008). Relationship between scratch hardness and yield strength of elastic perfectly plastic materials using finite element analysis. J. Mater. Res..

[19] Ye N, Komvopoulos K (2003). Indentation Analysis of Elastic-Plastic Homogeneous and Layered Media: Criteria for Determining the Real Material Hardness. J. Tribol..

[20] Johnson KL (1970). The correlation of indentation experiments. J. Mech. Phys. Solids.

[21] Zhang Y, Wang H, Li X, Tang H, Polycarpou AA (2017). A finite element correction method for sub-20 nm nanoindentation considering tip bluntness. Int. J. Solids Struct..

[22] Bhushan B, Li X (2003). Nanomechanical characterisation of solid surfaces and thin films. Int. Mater. Rev..

[23] Zhang Y, Polychronopoulou K, Humood M, Polycarpou AA (2017). High temperature nanotribology of ultra-thin hydrogenated amorphous carbon coatings. Carbon N. Y..

[24] Bowden FP, Tabor D (1966). Friction, lubrication and wear: a survey of work during the last decade. Br. J. Appl. Phys..

[25] Hamilton GM (1983). Explicit Equations for the Stresses beneath a Sliding Spherical. Contact. Proc. Inst. Mech. Eng. Part C J. Mech. Eng. Sci..

[26] Archard JF (1953). Contact and Rubbing of Flat Surfaces. J. Appl. Phys..

